# A field‐tested robotic harvesting system for iceberg lettuce

**DOI:** 10.1002/rob.21888

**Published:** 2019-07-07

**Authors:** Simon Birrell, Josie Hughes, Julia Y. Cai, Fumiya Iida

**Affiliations:** ^1^ Department of Engineering University of Cambridge Cambridge UK

**Keywords:** agriculture, learning, mechanisms

## Abstract

Agriculture provides an unique opportunity for the development of robotic systems; robots must be developed which can operate in harsh conditions and in highly uncertain and unknown environments. One particular challenge is performing manipulation for autonomous robotic harvesting. This paper describes recent and current work to automate the harvesting of iceberg lettuce. Unlike many other produce, iceberg is challenging to harvest as the crop is easily damaged by handling and is very hard to detect visually. A platform called Vegebot has been developed to enable the iterative development and field testing of the solution, which comprises of a vision system, custom end effector and software. To address the harvesting challenges posed by iceberg lettuce a bespoke vision and learning system has been developed which uses two integrated convolutional neural networks to achieve classification and localization. A custom end effector has been developed to allow damage free harvesting. To allow this end effector to achieve repeatable and consistent harvesting, a control method using force feedback allows detection of the ground. The system has been tested in the field, with experimental evidence gained which demonstrates the success of the vision system to localize and classify the lettuce, and the full integrated system to harvest lettuce. This study demonstrates how existing state‐of‐the art vision approaches can be applied to agricultural robotics, and mechanical systems can be developed which leverage the environmental constraints imposed in such environments.

## 1 INTRODUCTION

The story of agriculture is one of increasing automation. Crops are planted, weeded, and harvested with ever decreasing direct human involvement, reducing labor costs, and improving yield. However, every fruit or vegetable is different, and solutions for a single crop can vary from country to country and even company to company. While some crops such as wheat or potatoes have long been harvested mechanically at scale, many others such kiwi fruit (Scarfe, Flemmer, Bakker, & Flemmer, 2009), cucumbers (Van Henten et al., 2002), citrus fruit (Harrell, Adsit, Munilla, & Slaughter, 1990), strawberries (Hayashi et al., 2010), broccoli (Kusumam, Krajnik, Pearson, Cielniak, & Duckett, 2016), grapes (Luo et al., 2016; Monta, Kondo, & Shibano, 1995), and many others (Bac, van Henten, Hemming, & Edan, 2014) have resisted commercial automation. Agricultural robotics presents unique challenges compared to robotics in the more common factory environments (Oetomo, Billingsley, & Reid, 2009). Agricultural environments are unstructured, intrinsically uncertain, harsh on mechanical equipment (Reddy, Reddy, Pranavadithya, & Kumar, 2016) and have high variability over weather conditions, locations, and time. Autonomous agricultural systems must be flexible and adaptive (Edan, Han, & Kondo, 2009; Hajjaj & Sahari, 2016) to cope. Harvesting and other crop manipulation tasks (Hughes, Scimeca, Ifrim, Maiolino, & Iida, 2018; Kemp, Edsinger, & Torres‐Jara, 2007), are particularly challenging (Bac et al., 2014) along all these dimensions.

**Figure 1:**
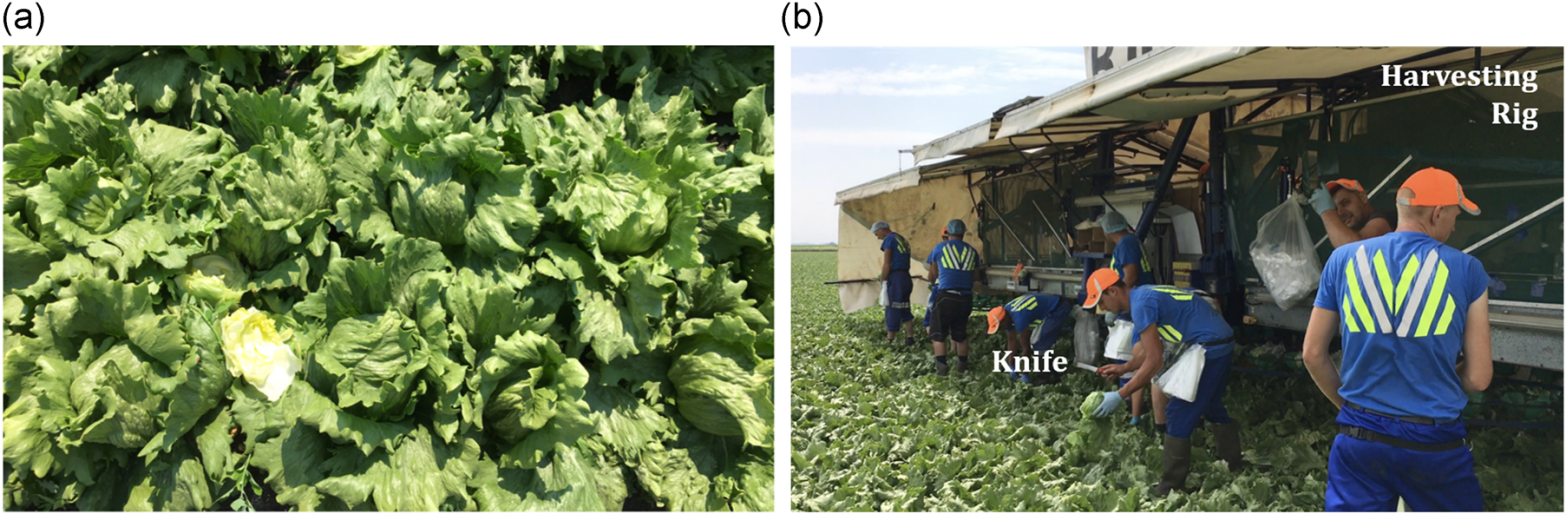
(a) The challenging localization and classification problem posed by the lettuce field. (b) The existing harvesting method [Color figure can be viewed at wileyonlinelibrary.com]

Iceberg lettuce is an example of a crop that is still harvested by hand using a handheld knife, and presents two main challenges to automation. First, visually identifying the vegetable's location and suitability for harvesting in what appears to be a sea of green leaves is hard even for humans (Figure 1a). Any solution must be robust to the variation in individual lettuces, with their appearance varying greatly over weather conditions, maturity and surrounding vegetation. Second, in a terrain with an uneven ground the lettuce stem must be cut cleanly at a specified height to meet commercial standards, while the lettuce head can easily be damaged by unpractised handling. A lettuce harvesting solution should therefore incorporate a high‐precision, high force cutting mechanism while being capable of handling the vegetable delicately. There is a growing need for automated, robotic iceberg lettuce harvesting due to increasing uncertainty in the reliability of labor and to allow for more flexible, “on‐demand” harvesting of lettuce (Bechar & Vigneault, 2016).

This study investigates automating the harvesting of iceberg lettuce with three key research goals. First, how vision systems can be developed using off‐the‐shelf convolutional neural networks (CNNs) as opposed to hand‐tailored computer vision pipelines, with pragmatic architectural adjustments made to allow for the data sets available. Secondly, how mechanical systems can be developed to work within the operational constraints imposed by the agricultural environment. Finally, how field robots can be developed to allow rapid integration and hence testing in the field.

This paper describes the results to date of the Vegebot project, where a lettuce harvesting robot has been developed using an approach of rapid iterative design, prototyping, and field testing. Two key methods are described for automating the harvesting of the iceberg lettuce under challenging and uncertain field conditions. First, the lettuces are localized and classified using a data‐driven approach. This is implemented using two CNNs, the architecture being shaped by the data sets available. Using this method in field tests, a visual‐based localization success of 91% in field tests was achieved, and the crop accurately classified. Second, the lettuces are harvested with a custom‐designed end effector that incorporates a camera, pneumatics, a belt drive, and a soft gripper. The end effector cuts the lettuce stems efficiently while grasping the lettuce head in a way that avoids damage. As the ground is uneven and its depth hard to detect under the foliage, a force‐feedback control system is used to detect when the end effector has reached the correct position to make the cut and achieve a consistent cutting height.

Following a review of the state of the art in crop harvesting, Section 3 defines the problem posed by iceberg lettuce harvesting and outlines the overall system that was developed. Section 4 focuses on the details of the two harvesting methods developed: the vision system and end effector. The field tests and experimental results are detailed in Section 5 and the paper concludes with a discussion and conclusion that suggests the application of the techniques and approaches in this study to other agricultural challenges.

## 2 STATE OF THE ART

There is prior work on vision techniques for agriculture. Many of the examples in the literature are from before the use of CNNs in the late 2000s, and so use a wide variety of hand‐crafted features. The detection of volunteer potato plants was performed using adaptive Bayesian classification of Canny Edge Detectors among other features (Nieuwenhuizen, Hofstee, & Van Henten, 2010). Broad‐leaved dock detection (a weeding task) was performed using a texture‐based approach, where image tiles were subjected to a Fourier analysis (Evert et al., 2011; weeding is a similar task to harvesting, just with less concern for the fate of the extracted plant). An alternative approach to weed detection used wavelet features of near infrared (NIR) imagery (Scarfe et al., 2009), subsequently passed to a principle component analysis (PCA) component and a *k*‐means classifier (Kiani, Azimifar, & Kamgar, 2010). Grapes have also been detected with Canny Edge filters, using decision trees as the classification mechanism (Berenstein, Shahar, Shapiro, & Edan, 2010). Foliage detection on the same project required a separate algorithm. Grapes were classified on another project using the AdaBoost framework, which combined the results of four weak classifiers into one strong one (Luo et al., 2016). Radicchios have been detected by thresholding hue saturation luminance images and applying particle filters (Foglia & Reina, 2006). Cucumbers were detected using NIR photography at two positions 5 cm apart, to give stereoscopic depth information (Van Henten et al., 2006) and classified for maturity by estimating their weight from the perceived volume (Van Henten et al., 2002). A more recent experiment detected broccoli heads using an RGB‐D sensor had the disadvantage that the robot had to move a tent across the field to prevent interference from outdoor light. Point clouds were clustered from the depth information, outliers were removed, and viewpoint feature histograms constructed. A support vector machine performed the actual classification of the broccoli heads (Kusumam et al., 2016). The use of vision to provide control through methods including visual servoing has also been shown to increase positional accuracy when harvesting citrus fruit (Mehta & Burks, 2014; Mehta, MacKunis, & Burks, 2016).

These solutions are not appropriate for iceberg lettuce. Color cues as used in (Berenstein et al., 2010; Cubero, Alegre, Aleixos, & Blasco, 2015; Foglia & Reina, 2006) are less useful because the lettuces appear to be a “sea of green.” Depth cues, as used in Kusumam et al. (2016) and Rajendra et al. (2008) also provide limited information because the plants and their leaves overlap and the heads are often hidden.

Similarly, there are a number of existing autonomous harvesting systems. Harvesting is a challenging task to automate and a recent review came to the gloomy conclusion that almost no progress had been made in the past 30 years (Bac et al., 2014). Many research projects have been performed, but little has filtered through into the commercial world. The more successful projects include a harvester for apples (Silwal et al., 2017) using a suction method, rice harvesting using custom harvesting systems (Kurita, Iida, Cho, & Suguri, 2017), and a sweet pepper harvesting system (Bac et al., 2017). There has also been significant work in the development of autonomous weeding or grading systems including a sugar beet classifying system (Lottes, Hörferlin, Sander, & Stachniss, 2017) and a grape pruning system (Botterill et al., 2017). There are a number of patents specifically relating to the harvesting of iceberg lettuce (Ottaway, 1996, 2009; Shepardson & Pollock, 1974); however, these have not been demonstrated under field conditions and do not clearly demonstrate how selective plant harvesting is possible. These previous approaches include using a belt‐driven band saw‐type mechanisms or water jet cutting. These approaches have limitations, most notably that the outer leaves of the lettuce can be easily damaged when harvesting and there is a lack of reliability in stem cutting height and quality.

## 3 PROBLEM DEFINITION AND SYSTEM ARCHITECTURE

### 3.1 Problem

The lettuces to be harvested must be both localized (their position detected) and classified according to their suitability for picking. For a mature lettuce, using the custom end effector, the lettuce head center must be localized to within approximately 2 cm of the ground‐truth position. The identified classes should include at a minimum (a) harvest‐ready lettuces (which may be picked immediately), (b) immature lettuces (which can be returned to later), and (c) infected lettuces (which should not be touched with the end effector so as to avoid spreading the infection). The vision system should operate under varying weather and lighting conditions.

Once a harvest‐ready lettuce has been identified it must be cut to supermarket standards. This is currently performed by a human worker with a knife. The worker tilts the head of the lettuce and then uses a high impulse maneuver to cut the stem of the lettuce. The lettuce is then bagged and placed on a harvesting rig (see Figure 1b). There is a high degree of dexterity and accuracy required to achieve a supermarket‐quality cut. The lettuce must have a stem of the correct length (1–2 mm protruding), and it must be clean, with minimal browning and have no damage to outer leaves. Additionally, if outer leaves remain after harvesting, these should be removed, which has proved to be a challenging manipulation problem in itself (Hughes et al., 2018). If the lettuce falls outside these requirements, it is not accepted by supermarkets. A lettuce worker can harvest a lettuce in under 10 s, which sets the benchmark for a robotic harvesting system.

There are also a number of constraints arising for the agricultural environment, which dictate the form factor and design decisions, and these are summarized in Table 1.

**Table 1:** Conditions for the design and development of a lettuce harvesting system determined by the agricultural environment

	Parameters	Specification	Influence on design
Environment	Width of lettuce lanes	2	Determines width of platform
	Spacing between lettuce	30 cm	Determines maximum size of end effector
	Height of lettuce plants	30 cm	Determines of height of platform
	Diameter of lettuce	20 cm	Determines size of end effector
	Diameter of lettuce stem	Approximately 30 mm	Determined blade specification
Robot	Generator power	240 V, 2 kW	Sufficient to power all systems
	Compressor air pressure	8 bar	Sufficient for pneumatics
	Vegebot dimensions	2 m x 0.6 m x 0.5 m	Fits within lettuce lanes

### 3.2 System architecture

**Figure 2:**
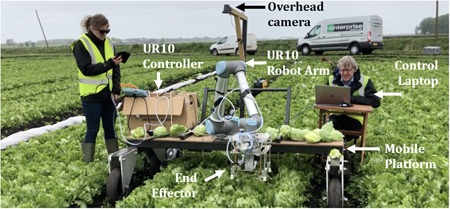
The Vegebot harvesting system, shown undergoing field experiments [Color figure can be viewed at wileyonlinelibrary.com]

**Figure 3:**
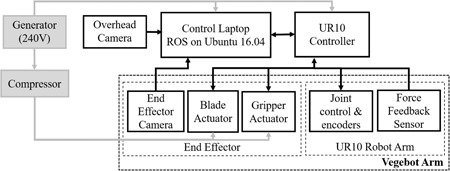
Block diagram of the robotic lettuce harvester system developed

The system developed for autonomous iceberg lettuce harvesting (Vegebot) is shown in Figure 2. Vegebot comprises a laptop computer running control software, a standard six‐degree‐of‐freedom (DOF) UR10 robot arm, two cameras, and a custom end effector, all housed on a mobile platform for field testing. A block diagram showing the integration of the system is shown in Figure 3.

Vegebot contains two cameras: an *overhead camera* positioned approximately 2 m above the ground and another *end‐effector camera* mounted inside the end effector. Both are ordinary, low‐cost USB webcams and stream video to the *control laptop*. Together, these allow Vegebot to detect (localize and classify) lettuces, and to move the end effector into position. There are additional sensors built into the *robot arm*: the standard *joint encoders* and a *force‐feedback sensor* that records the force and torque being applied to the end effector.

The UR10 arm provides a wide range of movements, and provides force and torque information allowing force feedback to be implemented. A commercial implementation would likely have simpler arms each with an end effector, all operating in parallel (for an example of such a system, see Scarfe et al., 2009). The control laptop controls the *end effector* using two digital I/O lines routed through the UR10 arm. These switch the two pneumatic actuators on and off, the *blade actuator* causing the blade to slice through the lettuce stalk and retract, while the *gripper actuator* causes the soft gripper to grasp and release the target lettuce.

The mobile platform supports the above hardware items and is moved manually around the field. The system is powered by a generator, which provides sufficient power to meet the peak demands of the system. An air compressor is used to enable actuation of the pneumatic systems. The generator and compressor can sit on the Vegebot to allow the system to be completely mobile.

**Figure B1:**
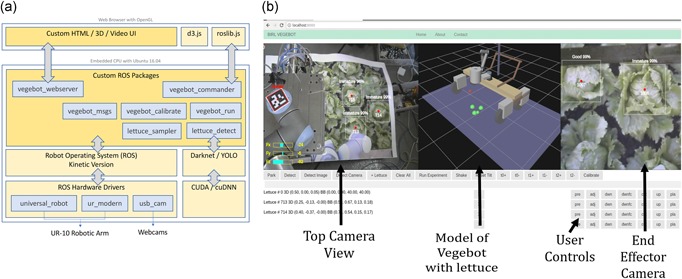
(a) The software architecture of Vegebot showing the structure and various packages used. (b) The web‐based user interface for Vegebot

The software architecture is shown in Figure B1a and detailed in Appendix B. The web‐based user interface is shown in Figure B1b.

#### 3.2.1 Control and processes

**Figure 4:**
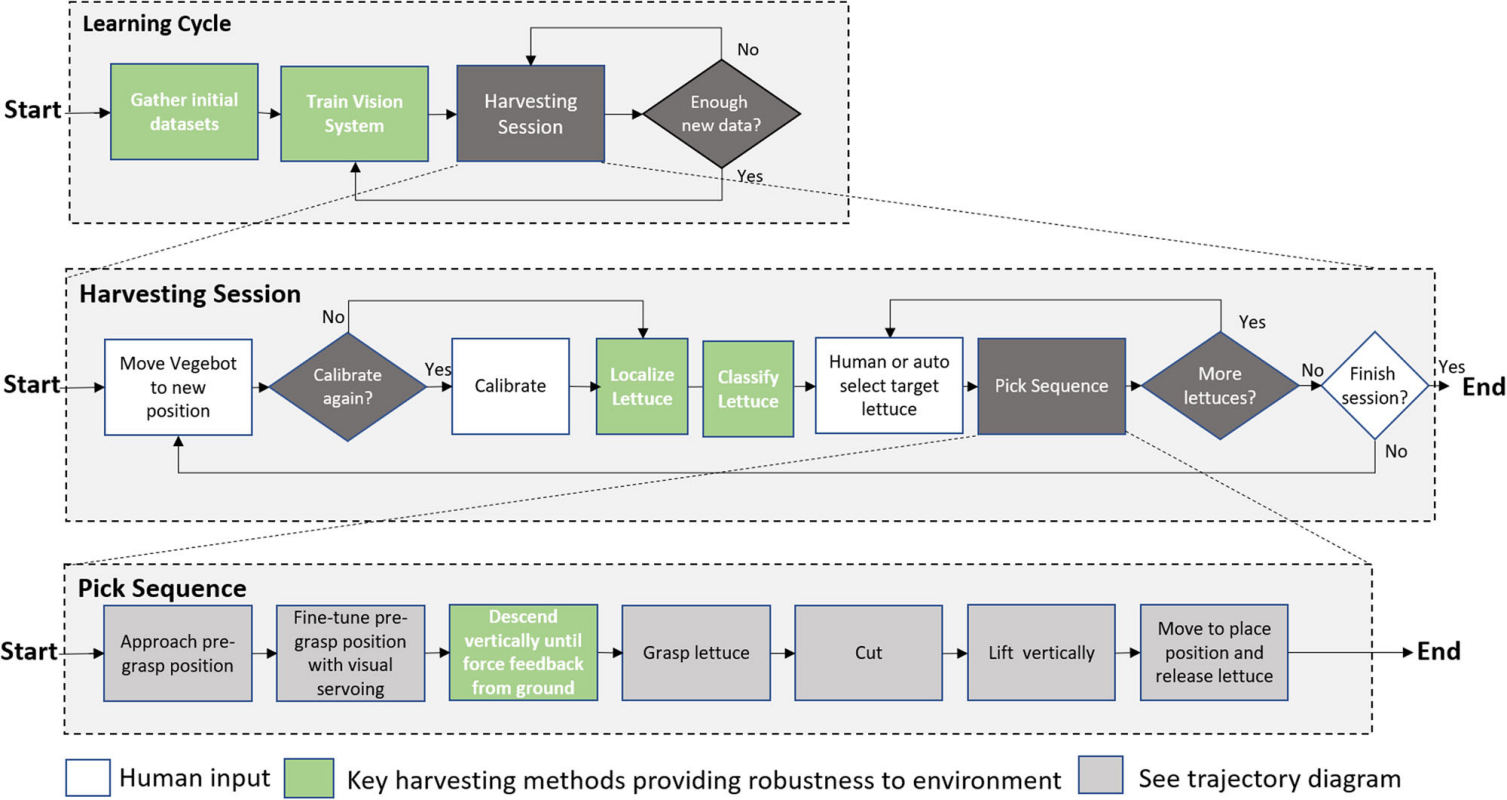
Processes for training and operation of the Vegebot, showing the key processes in green. The trajectory diagram for the lowest level pick sequence is shown in Figure 14 [Color figure can be viewed at wileyonlinelibrary.com]

The processes for training and operating Vegebot can be analyzed at three levels (see Figure 4). At the highest level, the *learning cycle*, data sets are gathered for the initial training of the vision system, harvesting is performed and additional data are gathered. As soon as enough new data are gathered to merit it, the system can be retrained. In this way, the accuracy and generalization abilities of the Vegebot can in principle be improved as images are obtained from new fields and under different weather conditions. The testing of these improvements is the subject of a future paper.

The *harvesting session* outlines the structure of the work in the field. First the Vegebot is moved along the lettuce lanes (seen in Figure 2) to bring approximately 10 lettuces within the robot's workspace and field of view. The current iteration of Vegebot is simply manually pushed into position. Next, the Vegebot is optionally calibrated, using the method described in Section 4.1.3. *Calibration* is always performed at the start of a session and then on an as‐needed basis as discrepancy between the lettuce position inferred by the overhead camera and that detected by the end‐effector camera increases.

Next, the vision system *detects lettuces* in the video feed from the overhead camera. A human then *selects a lettuce* by clicking on the user interface. This was a manual process during the experiments for the sake of safety. Selection could be automated with a trivial modification. The pick sequence then begins, with the lettuce being picked and placed onto the platform. Once the reachable lettuces have been picked, the Vegebot can either be moved to a new position or the session finished.

The *pick sequence* is fully automated and comprises seven stages. First, the end‐effector *approaches the pregrasp position*, a point centered approximately 10 cm over the inferred top of the lettuce, based on the localization predictions from the overhead camera. Because of the rugged nature of the environment and the impacts received by the Vegebot, this prediction is inevitably inaccurate to a greater or lesser degree. At this point, the camera in the end effector takes over to *fine‐tune* the end‐effector position to be directly over the center of the lettuce. The end effector then *descends vertically* down over the lettuce until the force‐feedback sensor registers the upward force of the ground resisting the downward trajectory. The soft gripper is then activated and *grasps the lettuce*. Next, the blade actuator is activated and the blade moves horizontally and *cuts* through the lettuce stalk. Still grasping the lettuce, the end effector then *lifts vertically* to the same height as the pregrasp position, clearing it from contact with the surrounding lettuces. The arm then moves the end effector to a convenient *place position* where the soft gripper is deactivated and the lettuce is released.

The following section addresses key the harvesting methods which have been implemented to allow robust and reliable harvesting in the agriculture environment (and are shown in green boxes in Figure 4).

## 4 HARVESTING METHODS

### 4.1 Lettuce localization and classification

The visual lettuce *detection* process comprises both *localization* (discovering where the lettuce is relative to the robot) and *classification* (determining whether the lettuce is a suitable candidate for being harvested). Lettuces heads are variable in appearance and are typically partially or wholly occluded by their own leaves and by leaves of neighboring lettuces. The outdoor lighting conditions also vary drastically with different weather, including very different levels of brightness and contrast. The lettuces need to be classified as “harvest ready” (for immediate picking), “immature” (for picking at a later date), or “infected” (to be avoided and reported). Additionally, the localization system must transform the viewpoint coordinates of the lettuce into robot‐centric coordinates for picking in the face of very rugged physical conditions. All these operations must be performed in close to real time given that Vegebot uses localization information dynamically to fine‐tune the trajectory of its end effector.

In principle, any of the latest deep‐learning based object detectors could fulfill this function. Candidates such as YOLOv3 and Faster R‐CNN (Redmon & Farhadi, 2018; Ren, He, Girshick, & Sun, 2015) can both provide object bounding boxes and class labels in real time (Ren et al., 2015). In this case, YOLOv3 was chosen as it gave the fastest detection times and its principal disadvantage (poor performance on very small close‐together objects) was irrelevant in this use case. Fast detection times on a laptop implied the possibility of later re‐implementing the algorithm on more modest, embedded hardware.

**Figure 5:**
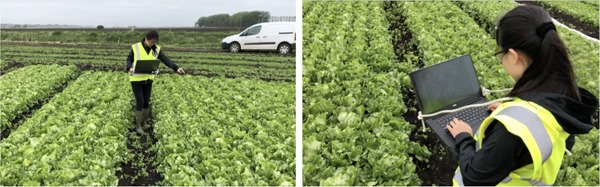
Obtaining data for the data set showing the user holding a webcamera to capture data sets at different heights [Color figure can be viewed at wileyonlinelibrary.com]

With a large enough detection data set, rich in examples of all lettuce categories, there would be little more to do. In the present project there were only two data sets available. The first was a detection data set gathered by one of the authors (see Figure 5), with images captured by a webcam and bounding boxes and class labels added manually. This data set (detailed in Table 2) was rich in positional data but the less common classes such as “infected” were underrepresented. The second data set originated from a previous student project (Nagrani, 2015, 1) in lettuce classification and was rich in examples of all classes, but had no useful positional information, all lettuces being in the center of each image.

**Table 2:** Details of the different sub‐data sets used to create the localization data set including the number of lettuce and conditions in which the images were taken

Sub‐data set	Number of images	Number of lettuce per image	Camera height from ground (m)	Weather conditions	Image quality
A	157	7–10	≈1.8	Cloudy/sunny	Medium
B	209	8–14	≈2	Sunny	High
C	117	3–6	≈1	Cloudy	Medium
D	131	4–11	≈1.2	Cloudy/rainy	Low
E	891	1	≈0.3	Cloudy/sunny/rainy	High

Ideally, a more extensive detection database would have been gathered from multiple fields and stages of the crop cycle, to fully represent the position and location of exemplars of all classes. Alternatively, the existing classification images could have been inserted over other backgrounds to produce an artificial training set for detection. This latter strategy runs the risk of the network learning to detect artefacts in the synthetic images, rather than genuinely localizing the vegetables based on natural visual cues.

**Figure 6:**
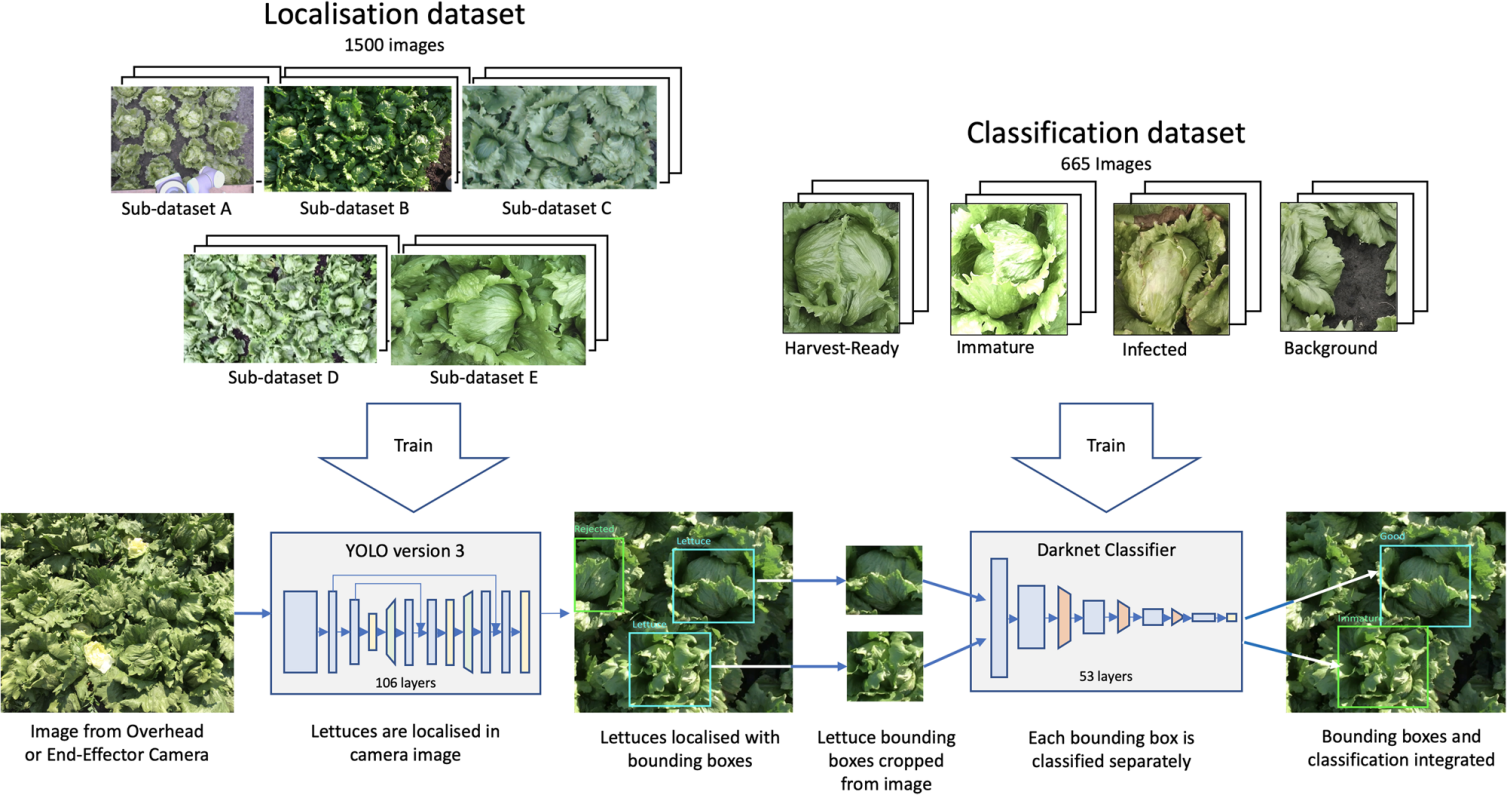
The vision system pipeline showing the two stages of convolutional neural network. First, the lettuces are localized using one network. A second network using both the lettuces localized from the first network and presegmented lettuce images from a classification data set is used [Color figure can be viewed at wileyonlinelibrary.com]

Instead, the solution chosen was to divide the pipeline into two networks (see Figure 6), each trained by one of the existing data sets. The first network, a YOLOv3 object detector would be used simply to discover the presence and location of lettuces (the number of classes being reduced to a single “lettuce” class) and output their bounding boxes. Narrow bounding boxes, likely caused by lettuces at the edge of the viewport and out of reach of the arm, are rejected as candidates. Each of the remaining bounded boxes is then cropped (adding a small margin round the outside of the bounding box to provide more visual information to the next stage) and then a second Darknet Object Classification Network was applied to each. Finally, bounding boxes predicted by the first stage and the class labels predicted by the second stage are merged. Although requiring a two‐stage network, this approach offers greater performance of both localization and classification. The architecture has been chosen to achieve the best performance with the data sets available and given the information content of those data sets.

There is an additional advantage to using a two‐stage network. Images input to YOLO are resized from 1,920 × 1,080 to a resolution of 320 × 320. This is still enough visual information to distinguish, say, a man from a dog, but may not be enough to determine whether one of the 10 lettuces visible in the overhead camera is infected or not. By first detecting the bounding boxes and then cropping each lettuce from the original 1,920 × 1,080 image before resizing to 224 × 224, much more visual information on each lettuce is available for the classification network. This improves the likelihood of a correct classification on images from the overhead camera.

Predictions on the network took 0.082 s for localization in the first stage and 0.013 s classification time for each detected lettuce passed to the second stage. Assuming 10 candidate lettuces per image the total time for localization and classification on the current hardware is approximately 0.212 s, slower than a single YOLO object detection network would be, but still sufficiently fast for real‐time adjustments. The end‐effector camera typically has only one lettuce in view during fine‐tuning, reducing the detection time to 0.095 s. The harvesting time is somewhat longer, and thus this is not the time limiting step. The pipeline processes images from both overhead and end‐effector cameras. The overhead camera provides candidates for picking and the end‐effector camera is used to fine‐tune the approach of the end effector to the desired lettuce.

The two‐stage network uses the existing data sets to maximum advantage and provides better classification by maintaining a higher resolution on the images of individual lettuces.

#### 4.1.1 Localization data set

Training a deep CNN object detector requires a large amount of data. The data set also needed to be a good representation of the real scenarios the Vegebot would encounter. Since there was no existing data set suitable for the propose of this project, a new lettuce localization data set was collected, labeled, and assembled. Images were collected from three different sources: images taken by the overhead camera on the Vegebot platform, images taken directly with a camera, and extracted images from videos taken by mobile phones and webcams. Figure 5 shows the process of obtaining images from the field using a webcam.

Images were divided into five sub‐data sets (A, B, C, D, and E) according to the characteristics of the images and corresponding to the different field experiments in which they were obtained. This allowed better tracking of the data set to make sure the assembled data set was well balanced. Figure 6 shows some sample images from each of the five data sets. The images cover different weather conditions, camera heights, lettuce fields, lettuce layouts, lettuce maturity, and image qualities, since these are factors that can vary during lettuce harvesting. Table 2 gives a detailed overview for each subset including the number of images, number of lettuces per image, camera heights, weather conditions, and image quality. Image quality refers to the subjectively evaluated blurriness of the images.

The images were labeled manually in square bounding boxes using the VoTT Visual Object Tagging Tool (Vott, 2018). The lettuce images were labeled such that center of the bounding box is the geometrical center of the corresponding lettuce and the dimensions of the bounding box are 10% larger than the lettuce head. Only the lettuces whose heads are fully included in the image were labeled. The data set was randomly separated into training (70%), validation (20%), and test (10%) sets, where the validation set is used for hyperparameter tuning and the test set is only used for benchmarking the final performance.

Even though only lettuces that were fully visible within the image were labeled, the YOLO algorithm was robust enough to detect lettuces at the edges as well. Classifying these partial lettuces would have increased the complexity of the problem unnecessarily. Practically, these lettuces were likely to be out of the reach of the Vegebot robot arm and therefore they were rejected from the detected candidates. There were also cases where lettuces were blocked by weeds, the Vegebot itself or other obstacles, which led to narrow bounding boxes instead of square ones. Lettuce rejection algorithms were implemented to reject such candidates. A candidate was rejected if it met either of the following criteria:

- Rejection of nonsquare bounding boxes which are on the edges of the images
  $$\frac{l}{w}>1.15\;\text{and}\;d<margin\;\text{where}\;\;margin=\frac{L+W}{75}.$$
- Rejection of narrow bounding boxes
  $$\frac{l}{w}>1.4,$$

where w and l are the lengths of the bounding box edges, with w being the longer of the two. *L* and *W* are the width and height of the overall image, and d is the distance between the bounding box and the edge of the image.

The localization network was based on the YOLOv3 architecture and was trained with a batch size of 64, subdivision of 8, and 10,000 iterations. The network was trained on a PC with a 4.5 GHz Intel i7‐7700k CPU and an nVidia 1080Ti GeForce GTX GPU. Training took around 12 hr. Pretrained weights based on ImageNet were used. No data augmentation was applied: This could improve localization performance and remains for future work.

#### 4.1.2 Classification data set

The goal of the classification network is to pick out the harvest‐ready (i.e., mature and healthy) lettuces among all the lettuces recognized from the previous localization step. Immature and infected lettuces should be left in the field. False‐negative localization results can be hazardous: Reaching for a nonlettuce object can damage the robot (if the object is a rock) as well as the object itself (if the object is a human hand or robot part). Adding a negative “background” class acted as an additional filter to prevent false positives: By explicitly labeling edge cases as not being lettuces, the classification network's performance improved.

The images were labeled by one of the authors with assistance provided by cultivation experts to allow labeling and classification of the data set. Figure 6 shows sample images from each of the four classes. Table 3 is an overview of the size of the data set. The 665 images were randomly separated into training (87.5%) and test (12.5%) sets.^2^ A higher portion of images were allocated to the training set deliberately due to the limitation of the images available.

**Table 3:** Classification data set, showing the number of each type of lettuce in the data set

Lettuce class	Harvest ready	Immature	Infected	Background	Total
Number of images	181	149	121	214	665

The classification network used was the standard object classifier supplied with Darknet, with no transfer learning (the use of pretrained weights would likely increase performance further). The batch size was 64, the subdivision was 4, and the network was trained to 260 iterations. The training was on the same hardware as the localization network and took 2 hr.

#### 4.1.3 Calibration and end‐effector positioning

The first approach tried on the positioning problem was the classic one of modeling the robot and its coordinate systems, calibrating the camera parameters, and then transforming the target center pixel of the lettuce (the center of the bounding box) to a position in 3D space and finally using inverse kinematics to move the arm as required. The problem encountered was that the system worked well in the lab, but would fail once subjected to knocks and bumps in the field. Even small deviations in the position of the overhead camera would mean that the robot might incorrectly locate its target by up to 10 cm.

A different approach was therefore attempted, where the robot could self‐calibrate the transformation from viewport pixels to arm position, using Aruco markers positioned on the top of the end effector. An occasional self‐calibration would be sufficient to reset the transformation, for example, after moving the platform. Calibration also resets the target location of the lettuce center within the viewport of the end‐effector camera. We assume the platform is kept approximately level with reference to the field due to the tracks in which them Vegebot moves. Further details of the final calibration procedure can be found in appendix.

### 4.2 Force feedback‐driven harvesting

The lettuce harvester has been designed to achieve reliable, efficient harvesting of lettuce with minimal damage to the lettuce. To meet supermarket specifications, the lettuce stem should be cut with a single consistent straight cut such that there is approximately 2 mm of stem. The outer leaves of the lettuce should also be removed where possible. A UR10 6‐DOF arm is used to provide movement of a custom end effector which has been specifically designed for lettuce harvesting. The UR10 arm is mounted on a mobile base which can be moved along the rows of lettuce.

The picking sequence (Figure 4 “pick sequence”) demonstrates how there are two stages to the physical cutting aspect of the harvesting procedure. To minimize the damage to the lettuce and also achieve a clean cut a method where the end effector is made of two mechanisms has been used. First, a soft clamping method is used to hold the lettuce throughout cutting and when lifting. Secondly, a cutting mechanism is required to cut the stem of the lettuce at a given height. The cutting mechanism requires force (≈20 N) to cut through the stem and outer leaves, while also requiring height adjustability and also a straight linear cut.

#### 4.2.1 End‐effector design

**Figure 7:**
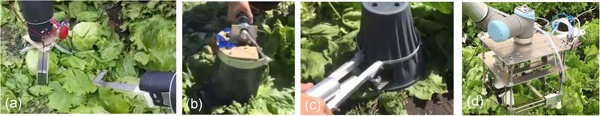
Development of lettuce harvesting end effectors. (a) Two‐handed approach with one hand to hold the lettuce, one hand with knife, (b) rotary DC motor cutting mechanisms, (c) linear actuator knife‐powered mechanism, and (d) pneumatic cutter chosen as the best mechanism [Color figure can be viewed at wileyonlinelibrary.com]

To achieve sufficient cutting force to cut the stem, a high impact, straight cut is required at the base of the lettuce. A number of different mechanisms were tested to determine which could achieve sufficient force and quality of cut: soft gripper and knife hand, pneumatic actuation, belt drive, and rotary chopping. Figure 7 shows the different mechanisms considered.

The two‐handed approach lacked sufficient cutting force and required a high level of coordination between the two arms. A rotary electric motor approach lacked the force to reliably cut the stem and led to the mechanism having to hack at the stem. Although the linear actuator approach provided sufficient force, the speed was low, leading to poor cut quality. The pneumatic cutting mechanics provides a high power‐to‐weight ratio, making it highly suited for this application where a fast clean cut is required. Although there is no position control, pneumatic actuation allows for easy to implement cut/open control.

The soft gripping mechanism has a single moving gripper and a fixed gripper lined with foam. Similar to other harvesting end effectors (De‐An, Jidong, Wei, Ying, & Yu, 2011; Foglia & Reina, 2006), a pneumatic actuator is used to control the gripper as this can be used to provide controllable compliance by varying the air pressure such that the lettuce is held but not damaged with simple open/close control

**Figure 8:**
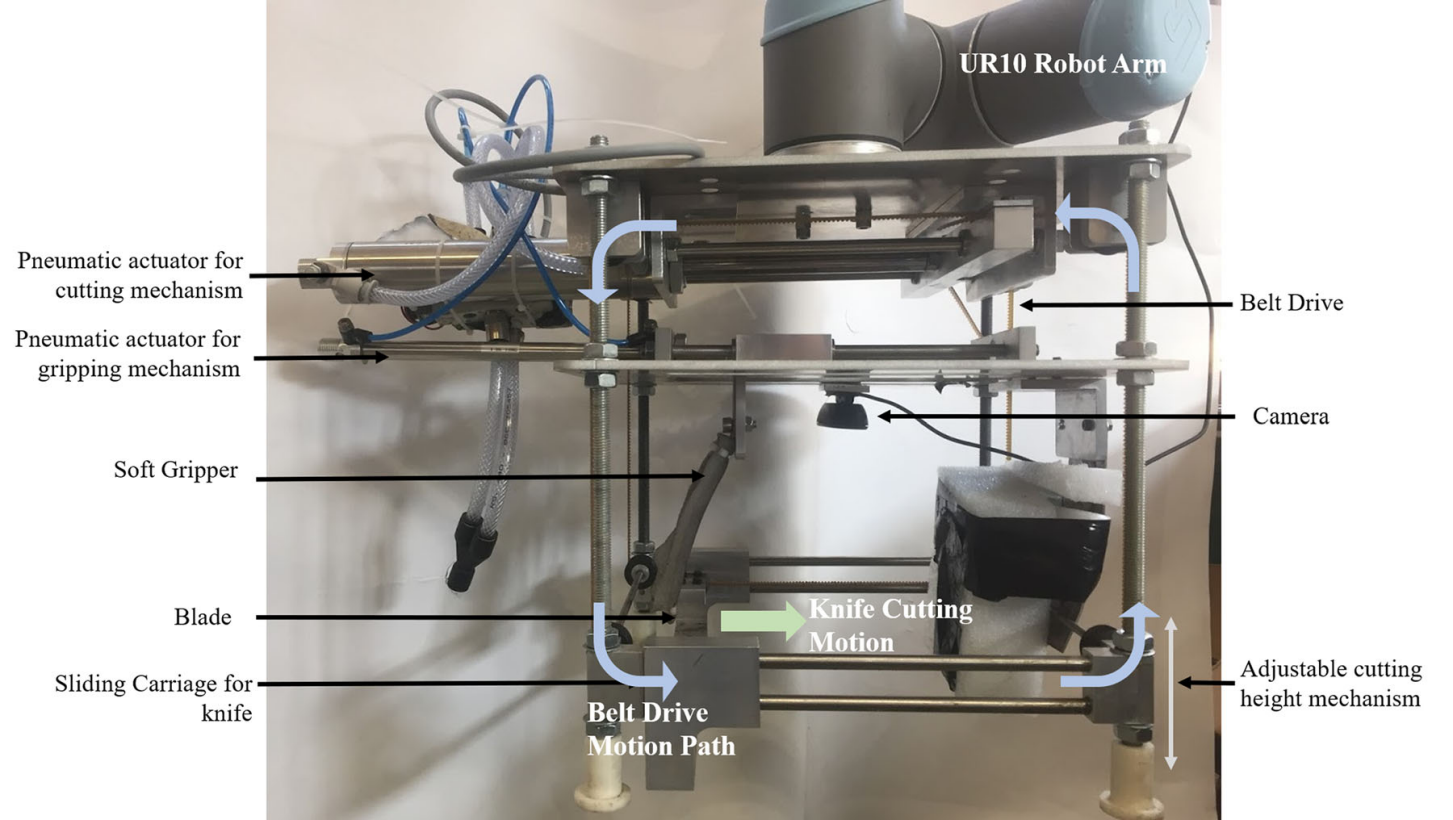
The final end effector developed, showing the belt drive mechanisms and dual pneumatic actuator system [Color figure can be viewed at wileyonlinelibrary.com]

The end effector developed is shown in Figure 8, with the design parameters given in Table 4. The end effector used only two actuators, one for grasping and one for cutting to enable simple control. A timing belt system was used to transfer the linear motion from a single actuator to both sides of the blade to allow smooth movement. This allows the actuator to be mounted above the height of the lettuce, such that when cutting it does not interfere. The belt drive system allows for the height of the cutting mechanism to be easily altered by changing the height of the cutting mechanism.

**Table 4:** Specification of the end‐effector developed

End‐effector parameters	Specification
Weight	8 kg
Height	45 cm
Width	45 cm
Depth	30 cm
Gripper pneumatic actuator specification	1 MPa, bore 10 mm, stroke 15 cm
Cutter pneumatic actuator specification	1.5 MPa, bore 15 mm, stroke 20 cm
Timing belt	5.08 mm pitch, 203 cm length, 20 mm width
Length of travel of blade	200 mm
Cutting knife length	250 mm
Inner area to encapsulate lettuce	25 cm × 25 cm

#### 4.2.2 Force‐feedback control

**Figure 9:**
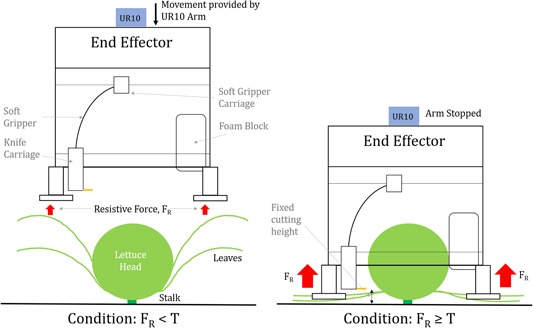
The force‐feedback method, allowing a repeatable height between the ground and the knife to be achieved [Color figure can be viewed at wileyonlinelibrary.com]

A key challenge to successful harvesting was reliably cutting the lettuce stalk at the correct height in an environment which is highly varying, uncertain, and unknown. To achieve this, the ground was used as a fixed reference point and the stem was assumed to be a fixed distance above the surface. Using force feedback from the joints of the UR10 robot arm, the end effector is lowered toward the ground, enveloping the lettuce, until a given force was achieved and contact with the ground could be assumed. The cutting height relative to the ground can be adjusted by manually varying the height of the cutting mechanism. A force threshold, T, was found by experimentally determining what force is required for the end effector to interact with the ground, that is, when it overcomes the resistive force of the leaves and other ground reaction forces, $F_{R}$. The force threshold was experimentally determined to be 60N to ensure all leaves were pushed away from the lettuce head and the end effector was in contact and level with the ground. This approach is summarized in Figure 9.

This approach helped push out the outer leaves of the lettuce which interfered with the cutting mechanism. This also allows the end effector to self‐level on the ground, and provided stability and consistency. Small “feet” were added to the end effector to allow stability to be achieved and prevent it from pressing too low into the ground. This approach allows the system to adapt to different field conditions, for example, different soil heights relative to the tractor track heights.

Once fully positioned, the lettuce is grasped and the cutting takes place. Each of the pneumatic actuators is controlled by a valve which has two position controls. Two digital outputs from the UR10 end effector are used to control the valves. After the correct height is achieved using force feedback, cutting is triggered by first actuating the grabbing mechanism so the lettuce is held in a fixed place. The cutter pneumatic system is then actuated so the blade cuts the stem of the lettuce. The arm can then be lifted, with the knife released and then the grabber retracted to release the lettuce.

Besides these two challenges, an additional one was that the weight of the end effector was at the limit of the payload ability of the UR10. This restricted the arm to moving more slowly than would otherwise be necessary. This will be discussed in the experimental results.

## 5 FIELD EXPERIMENT RESULTS

Ten experimental sessions were carried out in the harvesting seasons in 2016–2018 in lettuce fields in Cambridgeshire, UK, in varying weather conditions and across many (over 10) different fields. In these field trips, the system was developed and tested^3^.Field experiments were undertaken to test the performance of the localization and classification system in isolation from the harvester. The entire system was also integrated to test the full functioning of the system in conjunction with its physical harvesting abilities. In this section, the localization and classification is presented for both individual and system level tests, after which the harvesting system results are presented.

At the beginning of each experimental session, the Vegebot was assembled at the start of a lettuce lane. Typically, a three person crew participated, one operating the control laptop, one observer, and one checking and resolving any physical issues and enabling the air compressor when required.

### 5.1 Localization

**Figure 10:**
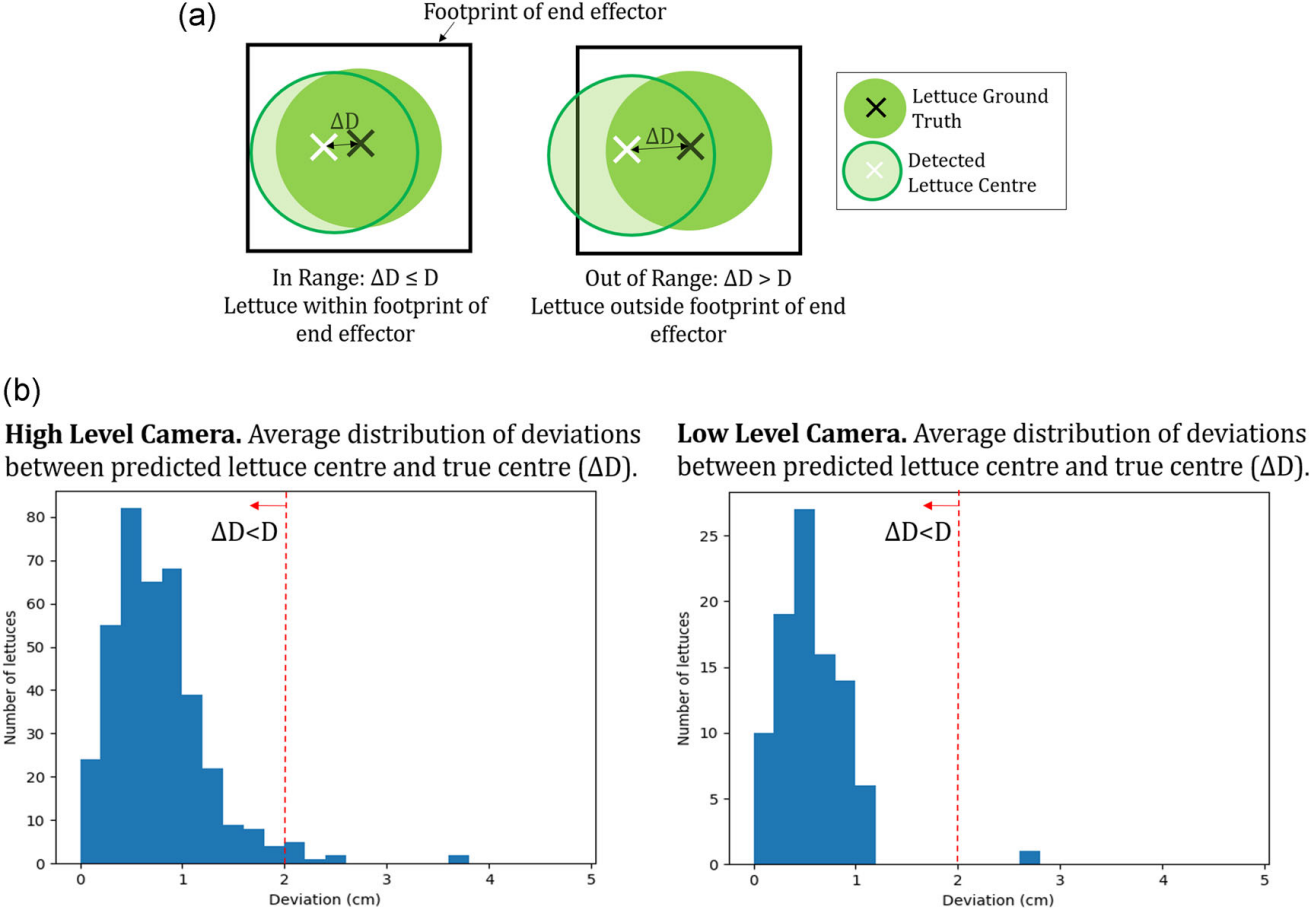
(a) The requirements for successfully lettuce harvesting determined by the physical end effector. The lettuce center must be detected within a distance such that the lettuce is fully within the footprint of the end effector when cutting. (b) The distribution of accuracy of the lettuce localization system for the two different cameras used, with images from sub‐data sets C and E, respectively [Color figure can be viewed at wileyonlinelibrary.com]

In order for a lettuce to be successfully picked, the center of the end effector must be placed with a tolerance, D, of the true center of the lettuce. The tolerance, D, which is determined by the mechanical design of the end effector is approximately 2 cm for average sized lettuce (approximately 15–20 cm diameter). For successful harvesting, the localization system must predict the center of the lettuce, such that the absolute difference from the ground truth, ΔD is less than the tolerance (ΔD<D). In practice, for a given camera height the threshold was specified in pixels, calculated taking into account the scale of the image. This threshold is illustrated by Figure 10a.

To test the ability of the system to localize lettuce heads with sufficient accuracy to allow success harvesting, images taken with both low‐level and high‐level cameras were used (approximately 30 and 170 cm above the crop, respectively). The difference between the detected and ground truth of the lettuce center was found. The distributions of the accuracy in the localization performance of the two cameras is shown in Figure 10b.

**Figure 11:**
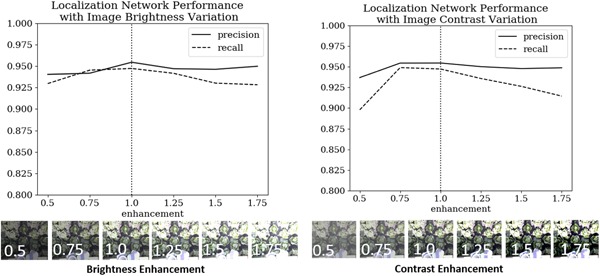
Localization performance with varying brightness and image contrast. The precision and recall are given in both cases. The images below show the contrast and brightness enhancement added applied to a typical image in the test data set [Color figure can be viewed at wileyonlinelibrary.com]

In the field, the lighting and weather conditions may vary significantly. To test robustness to different lighting conditions, the test subsets of data sets A‐E in Figure 6 were artificially modified with image processing (using ImageEnhance brightness and ImageEnhance contrast functions in the Python Willow library) to different levels of brightness and contrast, producing six times (7,200) the original number of test images (1,200). The localization system was then tested on this set of images (Figure 11). The precision and recall were then found. The system showed a high robustness to changes in image brightness (the most likely changing field conditions), with minimal changes in precision and recall. For the variation in image contrast, although the precision remained high, the recall dropped significantly for high changes in contrast. It is likely that using data augmentation techniques on the original training data set would have improved this.

**Figure 12:**
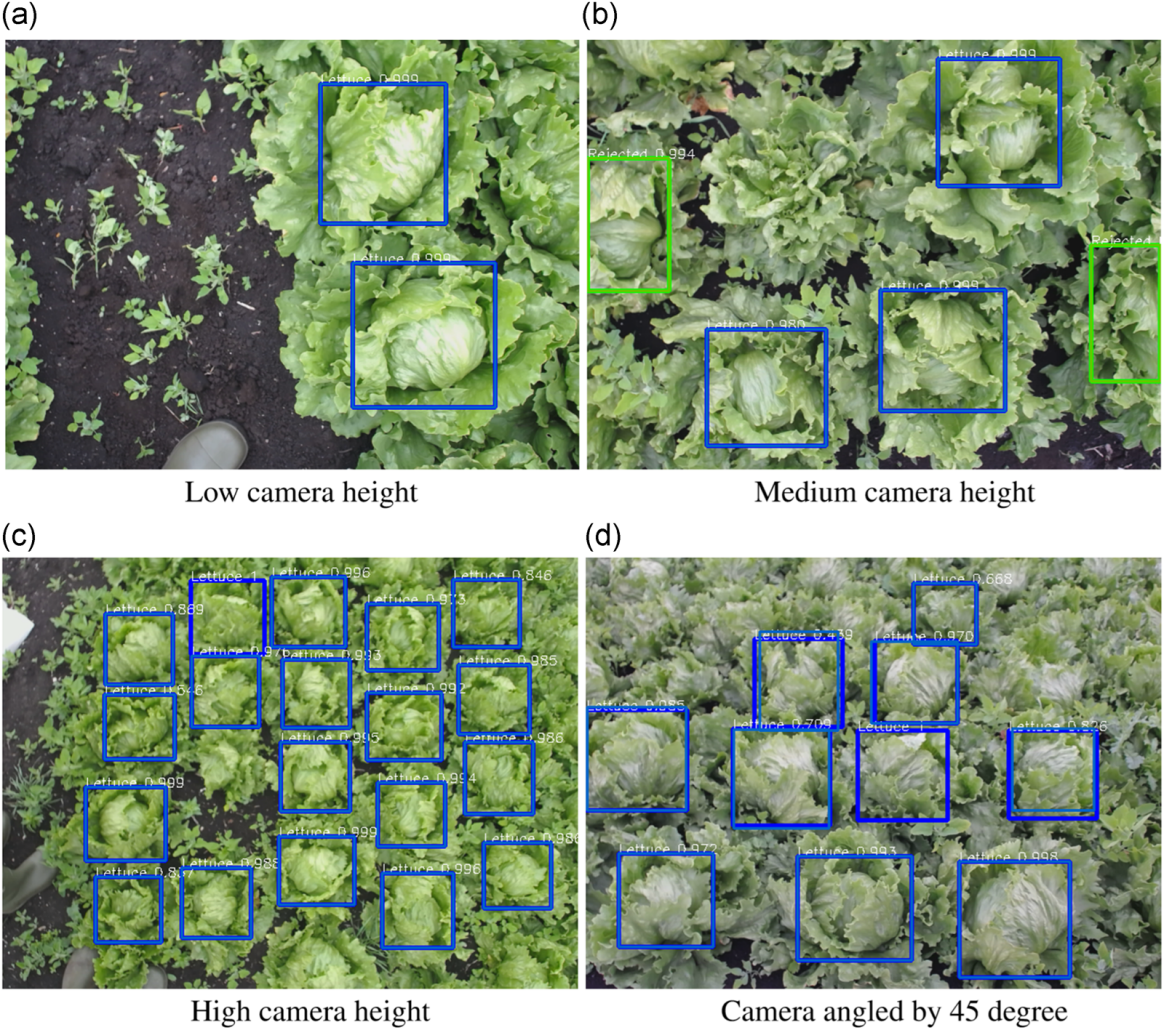
Examples of the localization system working on different lettuce and with camera setups with different heights and angles and showing usage on different crops and different fields demonstrating robustness. Blue bounding boxes indicate the entire head of lettuce could be seen, green indicate where only part of the head is visible [Color figure can be viewed at wileyonlinelibrary.com]

Figure 12 shows some examples of the localization results. Figure 12a–c shows the robustness at different camera heights, different angles (12d), and different parts of the field (middle and edges). The system was able to avoid detecting weed (12a,c), human feet (12a,b) as well as lettuces that fail to form lettuce heads (12b). Figure 12b also shows that the lettuce rejection algorithm is able to effectively reject lettuces which are on the edge of the image. Localization was also effective at different heights (ranging from 20 cm to 170 cm) and with the camera tilted by up to 45°.

When integrated into the full system, the overall performance of the localization system could be tested in harvesting trials. The success rate (number of correctly identified lettuce over total number of lettuce observed) and false‐positive detections were recorded. The results from this overall system results include over 60 individual lettuce harvesting experiments, where the localization results of all lettuce that could be visible observed by the system were recorded. The results are shown in Table 5.

**Table 5:** Overall system harvesting tests showing the localization performance

Metric	Result	Definition
Lettuce localization success	91.0%	$\frac{Number\;\;of\;\;detected\;\;qualified}{Number\;\;of\;\;real\;\;qualified}$
False‐positive detection	1.5%	$\frac{Number\;\;of\;\;false\;\;qualified}{Number\;\;of\;\;real\;\;qualified}$

### 5.2 Classification

**Figure 13:**
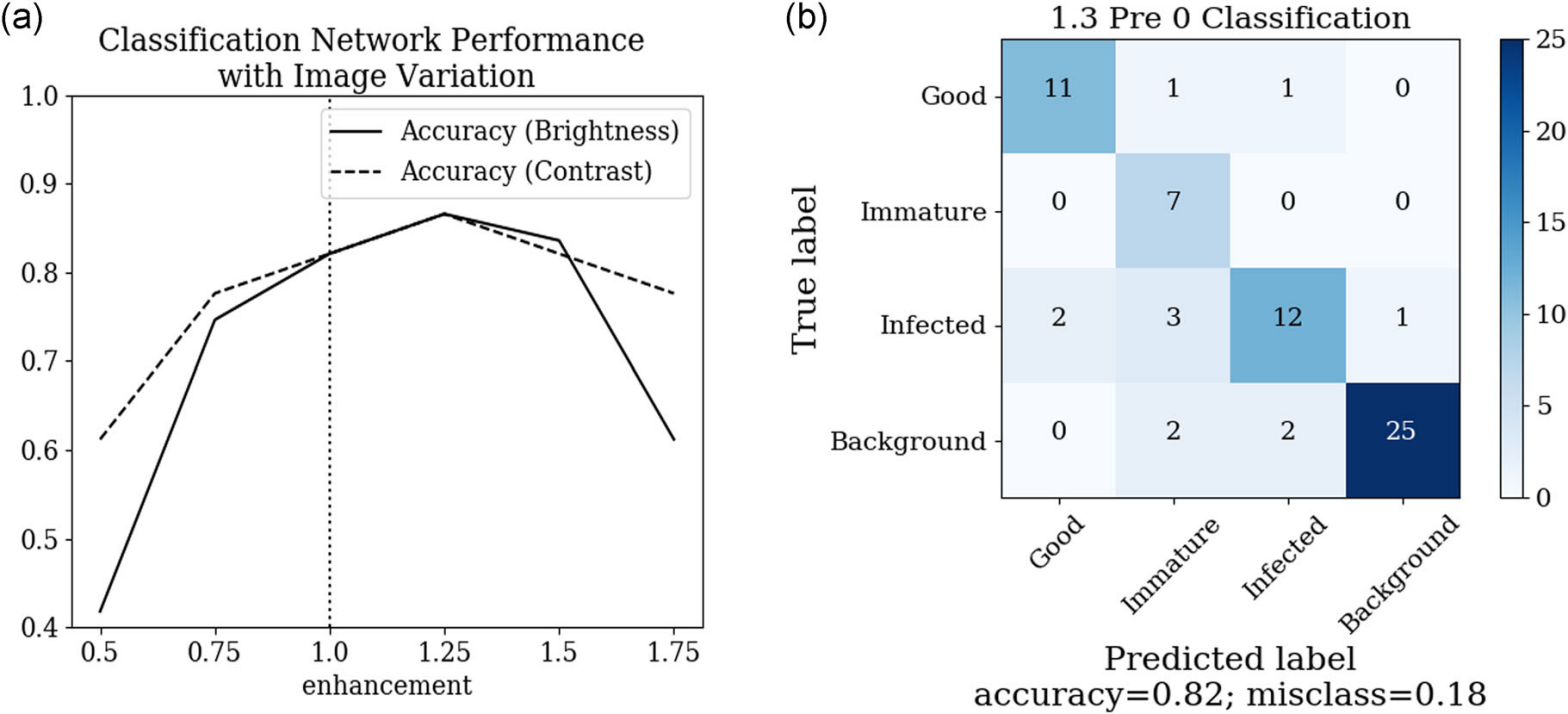
(a) Accuracy of the classification network with changes in image brightness and image contrast. (b) The confusion matrix showing the classification performance of lettuce [Color figure can be viewed at wileyonlinelibrary.com]

Robustness and accuracy of the classification system is critical for avoiding infected or damaged crops which could infect the harvesting system. By skipping immature heads and avoiding unnecessary harvesting the efficiency of the harvester can be maximized. To test the robustness of the system, the same images from the localization experiments (modified for brightness and contrast) were passed to the classification network and the accuracy recorded. The results are shown in Figure 13a. For classification, the network showed greatest robustness to contrast as opposed to brightness variations; this could be because the training data showed greater variation in contrast as opposed to brightness. Images taken in bright sunlight were high contrast rather than high brightness and there were no late‐night images in the data set to train for low brightness. Judicious data augmentation before training should improve performance.

To understand the classification decisions made by the network a confusion matrix of the field tests has been generated and is shown in Figure 13b. The diagonal shows the correctly classified lettuce, showing that the classification performs adequately for identifying background, infected and harvest‐ready lettuce. Identifying infected lettuce is crucial for avoiding contamination and further work should be undertaken to further improve the classification.

The network struggles to separate harvest‐ready and immature lettuces. One of the reasons is that the boundary between harvest‐ready and immature lettuces is very vague and changes accordingly to current market requirements, and thus creating a meaningful data set is challenging. The classification data set was labeled under the rules that a “harvest‐ready” lettuce head is around 18 cm in diameter, which for the majority of the time is the harvesting requirement. On the day of the field test, there was a change in harvesting specification: lettuces that would normally be treated as “immature” and left in the field were also harvested, which explains why many of the “immature” predictions got corrected to “harvest‐ready.”

When entire system tests of the Vegebot were later ran in the field, the system provide 100% accuracy when classifying lettuce. Although a reasonable number of experiments were ran (69), the number of nonideal (i.e., diseased or immature) lettuce in this experiment was low, so there was little variation in the classification of lettuce seen.

### 5.3 Harvesting performance

The final field tests were performed in May 2018 at a lettuce field in Cambridgeshire, UK. These final tests followed on from over 10 previous visits to the field with well over 300 lettuce harvested. The Vegebot was positioned at the start of a lettuce lane, the lettuces within the viewport of the overhead camera were detected and picks attempted. Once attempts had been made to pick all feasible lettuces, the platform was moved forward down the lane to the next unpicked rows. Each lettuce position, and false positives or negatives were recorded, together with the number and trajectory of all pick attempts. Finally, each lettuce was inspected for damage, in particular for the stalk being cut too close to the lettuce body. In total, 69 lettuces were detected by the vision system, 60 were in range of the robot arm and harvesting attempted with 31 lettuce harvested successfully. A video of the Vegebot in operation was recorded.^4^

#### 5.3.1 End‐effector trajectory

During the final field experiments, 69 qualified lettuces were detected by the vision system. Of these, attempts were made to pick 60, the remainder being out of range of the robot arm. Thirty‐one pick attempts were successful, with 29 failures, almost entirely due to the weight of the end effector causing mechanical failures on the arm which made attempting harvesting impossible.

**Figure 14:**
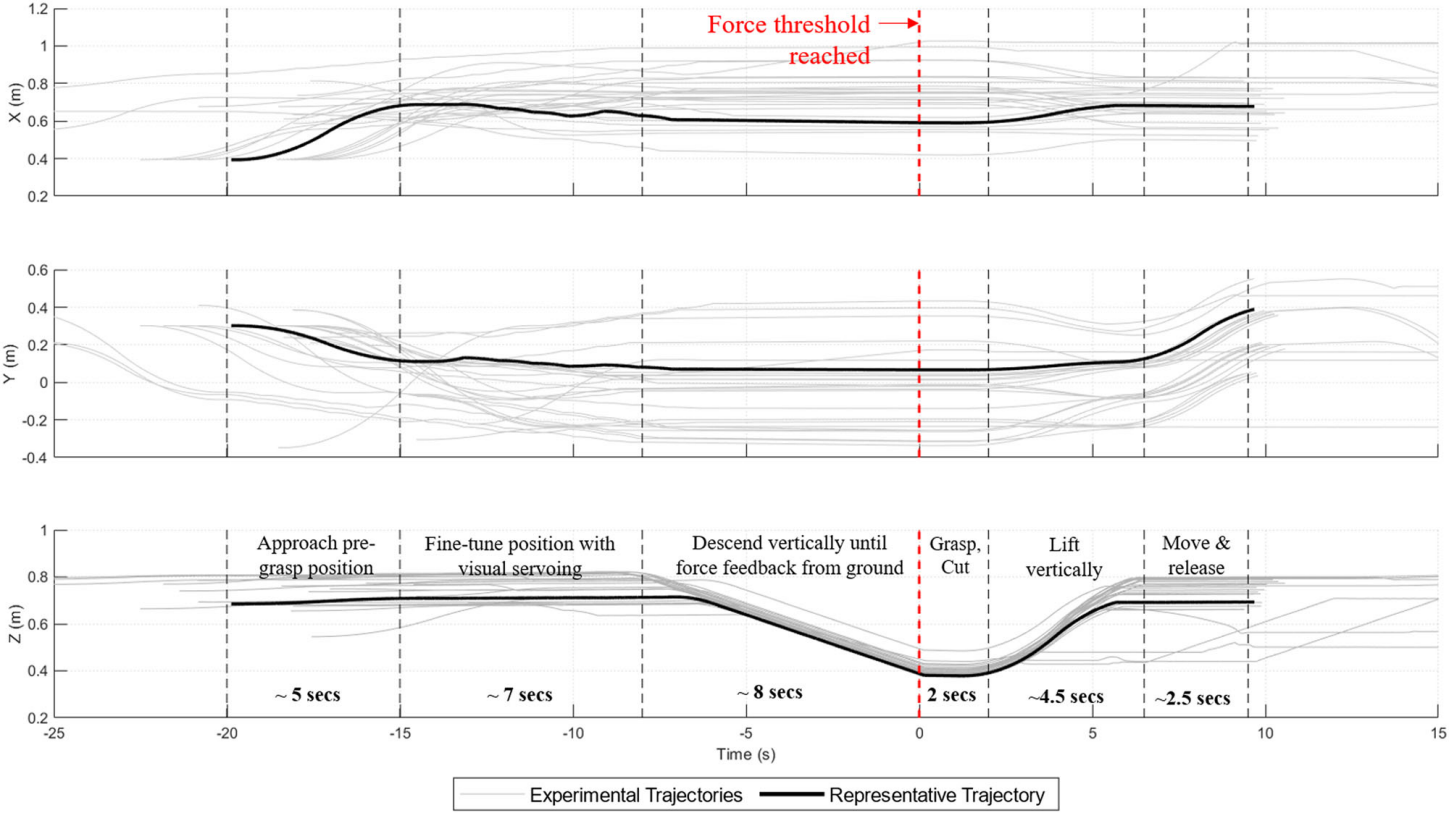
End‐effector trajectories when undergoing the field experiments. It shows all trajectories centered on cutting (at 0 s) and an example representative trajectory. The vertical divisions correspond to the different stages of the pick sequence from Figure 4 [Color figure can be viewed at wileyonlinelibrary.com]

The 31 successful trajectories of the end effector are shown in gray in Figure 14, with a representative trajectory highlighted in black. This representative trajectory shows a single experiment which reflects the desired trajectory and demonstrates the different parts of the harvesting process. The breakdown of the time series into the processes from Figure 4 is shown. The *X, Y*, and *Z* coordinates are shown with respect to the base of robot platform, with *X* pointing forwards in the direction of travel, *Y* pointing to the left, and *Z* pointing up.

With the exception of the *grasp‐cut* section, all of the other trajectory sections were slowed considerably by the burden of the end effector weight on the robot arm. This led to an average cycle time of 31.7 s. Critically, the rate‐limiting step, the grasping and cutting, required only 2 s. Thus, using a lighter end effector, for example, constructing from a lighter material such as carbon fiber, or using a stronger arm could lead to a significantly lower cycle time.

The trajectories clearly show the impact of the force feedback, with the robot arm descending in the *Z* axis at a consistent rate until the force threshold is met. This shows that the end height of arm varies considerably for different lettuce, showing how using force feedback allows a consistent height to be achieved. There is also slight variability in the *X* and *Y* axis close to when the force threshold is reached as the end‐effector self‐levels on the ground.

#### 5.3.2 Overall harvesting performance metrics

The results of the field experiments are shown in Table 6. Considering all the harvesting attempts, the detachment success if found to be 52% (31 out of 60 lettuces correctly identified, excluding false positives). However, in 28 cases, the harvesting failure was due to practical restrictions (weight of the arm, practical workspace of the robot arm, and the range of the overhead camera viewport), such that it was physically not possible to pick some lettuce. If the limitations of the arm are ignored, and the denominator reflects only those lettuces within the practical workspace, then the detachment success rises to 97% (31 out of 32). In other words, with one exception, if the arm could reach the lettuce, the end effector could pick it. Although this is a considerable exception, it could be simply achieved by using a robot arm with increased torque output.

**Table 6:** Overall system performance in the harvesting tests. Total lettuces attempted considers only lettuces within restrictions imposed by arm strength

Metric	Result	Definition
Total ground‐truth lettuces	69	
Total lettuces detected	61 (1 false positive)	
Total lettuces attempted	32	
Total lettuces detached	31	
Detachment success	97%	$\frac{Number\;\;of\;\;successfully\;\;picked\;\;qualified}{Number\;\;of\;\;detected\;\;qualified}$
Harvest success	88%	(Lettuce localization success) × (detachment success)
Cycle time	31.7 s, $\sigma^{2}=$ 32.6	Complete cycle time from lettuce to next
Damage rate	38%	$\frac{Number\;\;of\;\;lettuce\;\;harvested\;\;in\;\;unsaleable\;\;condition}{Total\;\;number\;\;harvested}$
Leaves to be removed	0.75, $\sigma^{2}=$ 1.42	Average leaves to be removed to achieve scalability
Total lettuces attempted	69	

**Figure 15:**
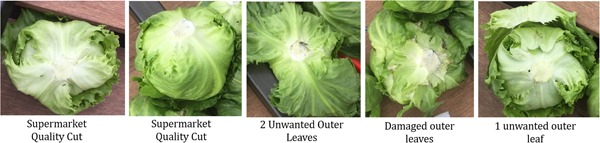
Examples of harvested lettuce showing some with an ideal cut, unwanted outer leaves and damaged outer leaves [Color figure can be viewed at wileyonlinelibrary.com]

**Figure 16:**
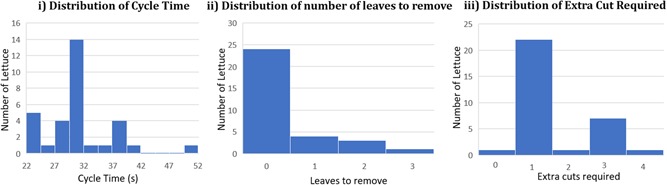
Distribution of the cycle times, leaves to remove, and extra cuts required for the various lettuce harvesting experiments [Color figure can be viewed at wileyonlinelibrary.com]

Examples of the harvested lettuce are shown in Figure 15, showing high‐quality cuts and also showing those with unwanted outer leaves or damage. The distribution of the lettuces which required extra leaves to be removed, extra cutting attempts and the cycle time is shown in Figure 16. The cycle time varies greatly depending on how far the arm needs to travel from lettuce to lettuce, exacerbated by end‐effector weight slowing the movements. In a few cases, one extra leaf needed to be removed (manually) to achieve supermarket perfection. Additionally, in some cases extra cuts were required. This was often due to the leaves of the lettuce and movement of the lettuce head within the cutting area. Additionally, the cuts were generally a little too close to the body to be acceptable in the current market.

The average cycle time was 31.7 s, with a variance of 32.6 s. Again, this value was largely due to the limitations of the arm and the weight of the end effector. Of the trajectory sections in Figure 14, all but the short grasp‐cut section (2 s) have their speed limited by the arm's payload capacity. A much reduced cycle time should be achievable with a stronger arm or lighter end effector. In addition, around a quarter of the cycle time is taken by the fine‐tuning of the end‐effector position. Any improvements to the accuracy of the overhead camera localization would further reduce the overall cycle time.

Reducing the damage rate (38%) will require further experimentation. Supermarket chains, the largest wholesale lettuce buyers, have strict standards for the length of the cut stalk to improve the vegetable's appearance in packaging. According to these standards, esthetic rather than relevant to the lettuce's suitability for eating or not, the end effector often missed the ideal length, cutting in most cases slightly too close to the lettuce head. Of the 32 picks, only two actually resulted in inedible lettuces. Improvement can probably be made by refining the force‐feedback mechanism and perhaps introducing field‐dependent depth calibration at the start of each session. This remains for future work.

Again, buyer standards dictate that a packaged lettuce should not have too many superfluous leaves in the packaging. At present, a human harvester will deftly remove a few leaves after each pick before passing the lettuce onto the harvesting rig. The end effector left the picked lettuce with an average of 0.75 additional leaves that are undesirable by these standards. These would have to be removed further down the production chain by hand, or in an automated fashion.

It is worth noting that both the metrics for damage rate and leaves to be removed could be substantially improved by permitting a greater range of appearance of the vegetable on supermarket shelves. Until the robot improves, this suggests a dual pricing strategy, with a higher price paid by the consumer for a “perfect” hand‐picked lettuce and a lower price for a more variable but quite edible robot‐picked one.

## 6 DISCUSSION

There is much remaining work required to achieve an iceberg lettuce harvester for commercial operation. Existing challenges include visual analysis, precise manipulator control, harvesting rig development, and reduction of the overall cycle time and costs. In this study the focus was not to develop a commercial product, but to demonstrate proof‐of‐concept experiments which provide research outcomes which can aid future development of agricultural robotics systems not only for iceberg lettuce, but many other crops. This section discusses the design rationale behind the development process and in particular the visual processing strategies which were chosen and how these approaches can be used to aid future work in this field.

The final prototype of Vegebot is a result of more than 15 iterations and on‐site field tests which were carried out in the UK harvest seasons (July–September) between 2016 and 2018, and also countless lab based experiments. In each iteration, new software and hardware redesigns were tested in the field, data gathered, and results compared. The development approach adopted was to produce a modular system to enable rapid integration and testing of the architecture systematically. Frequent field tests were used to provide feedback and to identifying the improvements required. As a consequence of this approach, the physical design changed radically from week to week (see Figure 7). This process was kept grounded by the use of standard harvesting metrics (Bac et al., 2014) to monitor progress. The authors believe that this iterative approach is more likely to yield robust, field‐worthy robots than careful upfront design based on an idealized version of the problem.

As an example of the approach taken, the available visual data sets of lettuces were not ideally suited for an optimal vision system. Two separate data sets, one for localization and one for classification, were both of reasonable quality in themselves but in an ideal world would have been combined into one integrated whole. Rather than spend time and resources gathering yet another data set to replace them, the Vegebots neural networks were quickly adapted to make use of what was available. This enabled the robot to detect lettuces correctly, solving the problem for the time being and allowing work on the overall system to continue. With future iterations and online data‐gathering this architecture could be simplified once again into a single, fully‐integrated CNN architecture.

It is noteworthy that a vision system based on a standard CNN architecture was able to achieve the localization results that it did, given the difficulty of the task for a human harvester. Many of the previous harvesting robots detailed in Section 2 required vision systems carefully tailored to the fruit or vegetable in question (e.g., detecting color or depth). For example, broccoli heads are detected using an elaborate pipeline of RGB‐D sensors, point clouds, and feature extraction in Kusumam et al. (2016) and radicchios using hand‐crafted features and particle filters in Foglia and Reina (2006). CNNs, together with some rapid and informal data gathering, proved “good enough” for the nontrivial localization of iceberg and may turn out to be sufficient for other crops (Kamilaris & Prenafeta‐Boldú, 2018).

Considering the mechanical development, by making field testing central to the project, the robot design naturally adapted itself to real‐world commercial conditions. Vegebot operates in the same fields and along the same lane layout as human harvesters. Neither the environment nor the crop itself was altered in any way to facilitate the automated harvesting. By contrast, solutions using water knives require careful selection of the crop variety and modifications to the way they are planted (Simon, 2017). Vegebot‐derived solutions could be gradually deployed alongside existing methods, rather than requiring major changes to existing practices. The control and calibration software was repeatedly simplified to provide a solution that worked robustly in the field. Sensors were stripped out, not added. Complex algorithms to model in 3D and determine the optimal cutting position were replaced with mechanical legs that provided force feedback from the ground, giving the robot a simple signal on when to cut. A design change was considered an improvement whenever a mechanical feature or software module was eliminated. In the long term, this preference for simplicity over sophisticated solutions may prove limiting, yet Vegebot has already achieved important results. The use of standard metrics as proposed by Bac et al. (2014) kept the project on track and focused on steady, incremental improvements. The authors feeling is that the iterative, simple approach can yield yet many more dividends before being exhausted.

As the project stands, the *damage rate*, caused by cutting the lettuce stem too short, is too high for supermarket standards, although the harvested vegetables were perfectly edible. The most recent sample size of 69 lettuces was enough to confirm this as the next problem to address (hundreds of lettuces had been harvested over previous iterations). Future versions of Vegebot will need to address and improve the damage rate, perhaps with visual feedback from the harvested lettuces dynamically adjusting the force threshold at which the cut is made. In parallel, the end effector needs to be made lighter to achieve a human‐level *cycle time*, possibly by manufacturing with carbon fiber, or by using an alternative, stronger cartesian arm design.

In summary, the adaptation of CNNs to pre‐existing data sets and the use of simple, low‐sensory, environmental feedback may prove useful in other harvesting projects. The authors key recommendation would be rapid iteration with radically different hardware designs, testing in the field as often as possible and relentlessly simplifying and using the standard metrics to stay on track.

## 7 CONCLUSIONS

This paper presented a proof‐of‐concept platform called Vegebot that demonstrated an automated and potentially autonomous approach to harvesting iceberg lettuces. The vision system, mechanics, and control strategy were described and the experimental results detailed.

The goals of the project were to achieve a robust localization and classification, to achieve a cycle time comparable to humans and to avoid damage to harvested lettuces. The localization and classification were reasonably robust, as demonstrated by a localization success of 91% and a classification accuracy of 82% when tested on a significant test data set. The average cycle time on Vegebot (31.7 s) was restricted by the weight of the end effector and thus currently slower than humans, but could be easily improved in subsequent versions made from lighter materials. Although the harvest success rate was high (88.2%) the damage rate was poor (38%). The sample size of 60 lettuce demonstrates potential and identifies that future work is required to reduce the damage rate. Further optimization is required to meet supermarket standards.

In comparison with other work in this study ecosystem, we have demonstrated a number of new approaches and techniques for agricultural robotics. In using a two‐stage CNN we have used an “out‐of‐the box” learning system for a specific agricultural problem as opposed to creating a bespoke system for this particular problem. This is different from many state‐of‐the‐art solutions (Berenstein et al., 2010; Ren et al., 2015). We have also explored how this approach can make best use of the available data sets and can implement full data collection, training, and testing. Additionally, in the development of the mechanical components of the harvesting system we have shown how the environmental constraints can be exploited. This has been shown to help achieve a consistent cutting height. This use of the environment, and designing mechanical systems to work within an existing agricultural environment, is different to many other approaches. This presents an approach to achieve robustness in challenging agricultural environments.

While the immediate future would appear to be robot arms attached to harvesting rigs, an autonomous Vegebot is also a distinct possibility. While its capacity would clearly be more limited, it would have agility in the sense of responding quickly to sudden spikes in demand. Marshaling a human team and a harvesting rig can be difficult at short notice and may be overkill for unexpected but smaller orders, whereas an autonomous Vegebot could be conveniently sent into the field to fulfill them. Outside of harvesting time, it could also be used for data gathering. The vision and learning system in combination with the end‐effector system provides the potential for selective plant harvesting. This could increase crop and harvesting efficiency.

Agriculture is an industry where margins are low; cost efficiency and time efficiency are key. To make the presented approach viable, the cycle time would need to be reduce to that comparable to humans. However, using a robotic system would enable certain advantages such as a more flexible work force and nighttime operation. The techniques and approaches here have been applied to iceberg lettuce; however, the concepts could be applied to other harvesting and robotic agriculture situations. Further work to investigate wider applicability, and developing a more universal harvesting system would increase both commercial and research impact.

## ACKNOWLEDGMENTS

This project was possible thanks to EPSRC Grant EP/L015889/1, the Royal Society ERA Foundation Translation Award (TA160113), EPSRC Doctoral Training Program ICASE AwardRG84492 (cofunded by G's Growers), EPSRC Small Partnership AwardRG86264 (in collaboration with G's Growers), and the BBSRC Small Partnership GrantRG81275. In addition, we are extremely grateful from the support and valuable time input from G's Growers, in particular Charlie Kisby, John Currah, James Green, and Jacob Kirwan. We would also like to thank Dr. Alex Jones from the Sainsburys Laboratory and many who have contributed to the iterations of Vegebot: Luca Scimeca, Andre Rosendo, Fabio Giardina, Claudio Ravasio, and Vivian Wong.

## Figures and Tables

**Figure C1:**
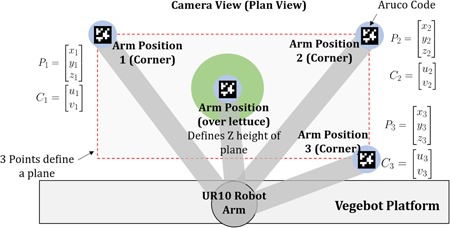
Calibration method, showing how position and camera coordinates are gained from three positions to allow a mapping from camera to real‐world coordinates to be achieved

## Footnotes

1
http://mi.eng.cam.ac.uk/projects/lettuce/

2 The Darknet classifier has no separate validation data set; the experimenter chooses the length of training based on periodically evaluating against the test set. For the robustness evaluation below, fresh data was used.

3 These were in collaboration with a major agricultural company, G's Growers.

4
https://youtu.be/UR-7LBdI7Z4

## APPENDIX A INDEX TO MULTIMEDIA EXTENSIONS

**Table:**

Extension	Media type	Description
1	Image	Overhead view of lettuces
2	Image	A lettuce harvesting rig with workers
3	Image	The Vegebot lettuce harvesting robot
4	Image	Block diagram of Vegebot
5	Image	Process diagram of Vegebot
6	Image	Scientist gathering data in lettuce field—two photos
7	Image	Image pipeline of Vegebot
8	Image	Four photos of four end effectors
9	Image	Labelled photo of final end effector
10	Image	Diagram of how end effector works
11	Image	Overhead diagram of end effector positioning over lettuce
12	Image	Distribution diagram
13	Image	Two line graphs with photos below
14	Image	Four photos of lettuces with bounding boxes
15	Image	Line graph
16	Image	Confusion matrix
17	Image	Diagram of trajectories
18	Image	Five photos of lettuces
19	Image	Three distribution graphs
20	Image	Software architecture
21	Image	User interface
22	Image	Calibration diagram

## APPENDIX B SOFTWARE

The software (see Figure B1a) was written on the kinetic release of robot operating system (ROS). Custom ROS modules for Vegebot were written in Python and are bundled as the package vegebot^5^:

- vegebot_commander: This node is responsible for receiving user commands from the web‐based user interface front‐end and either executing them or passing them to the appropriate node.
- lettuce_detect: This node encapsulates the code that classifies and localizes lettuces from a 2D image. It calls the two deep neural networks running on Darknet.
- lettuce_sampler: This node supplies sample 2D lettuce imagery for testing purposes when not in the field.
- vegebot_msgs: This node defines the custom ROS messages used for internode communication, including lettuce hypotheses.
- vegebot_webserver: This node serves the HTML front‐end user interface to the robot operator.
- vegebot_run: This module contains the 3D model of the Vegebot (in URDF format) and the scripts for launching the entirety of the software under different conditions.

Standard ROS hardware drivers (universal_robot, ur_modern, and usb_cam) are used to drive the UR10 arm and the webcams. A standard installation of Darknet (Redmon, 2013) with YOLOv3 was accelerated by CUDA drivers version 9 to provide image detection services. The HTML user interface (see Figure B1b) can be operated on the same control laptop or remotely, via an onboard WiFi router. The two cameras stream live video to the user interface and bounding boxes and classes for the detected lettuces are overlaid. The position of the calibration marker is also shown. The roslib.js library provides an interactive 3D model of the robot which displays the real robot's movements. The force feedback on the end effector is shown by three bar graphs to the left of the display. Detected lettuces are added dynamically as menu items to the screen, using the d3.js library. The operator can test individual actions (such as “move to pregrasp position”) or simply select a detected lettuce and instruct Vegebot to pick and place it.

## APPENDIX C CALIBRATION DETAILS

The full calibration sequence was as follows and is summarized in Figure C1.

1. Manually position the end effector over any lettuce *X* using standard UR10 controls.
2. Manually raise the end effector vertically until approximately 10 cm clear of the lettuce.
3. Trigger automatic calibration:
  (a) The center pixel of the bounding box for lettuce *X* in the end‐effector camera is recorded as the target center pixel for fine‐tuning (the camera is not centered in the end effector for space reasons)
  (b) The calibration records the vertical position of the end effector (*Z* axis in ROS) and assumes this to be the height of the plane containing all future “pregrasp” positions.
  (c) The end effector then moves to three positions at the edges of the viewport, in the same horizontal plane. Each position is recorded in terms of the *X, Y, Z* of the end effector in the robot arm's coordinate frame and in terms of the *u,v* center pixel of the detected Aruco marker.

The three calibration positions define a horizontal plane with respect to the ground, around 10 cm over the tops of the lettuces. Given any pixel *u,v* in the viewport, the corresponding *x, y, z* in the horizontal plane can be found by linear interpolation between these three points. The UR10's built‐in inverse kinematics were then used to move the end effector into position in the “approach pregrasp position” phase of the pick sequence (see Figure 4). For further details of the calculations, see Appendix C.

This rough positioning proved robust enough to move the end effector into the pregrasp position, but not to exactly center it accurately over the top of the lettuce. At this point, the end effector “fine‐tunes” the position using a simple visual servoing method. The bounding box of the target lettuce is now visible in the end‐effector video feed (see Figure B1b, right‐hand video feed for an example), the center point is calculated and then the arm is moved in the horizontal plane (along the *X* and *Y* axes) until this center point coincides roughly with the target pixel recorded in Step 3a of the calibration sequence. The end effector is now positioned over the center of the target lettuce and can then descend vertically.

While the full calibration sequence involves human input to position the end effector over a sample lettuce, the resampling of the horizontal plane itself is automatic and could be triggered without human intervention on an as‐needed basis, for instance when the ‘fine‐tuning’ phase of the trajectory starts to take too long or to fail.

The calibration procedure is always undertaken when the Vegebot is positioned at the start of a lettuce lane. When the platform is manually moved between harvesting sessions, there is a human decision (see Figure 4) on whether recalibration is required, if for example the change in terrain has caused the relative position of the platform to the field to change. This can be seen in the increasing amount of time taken to fine‐tune the end‐effector position. Long term, this process would be automated.

Three calibration points in robot space (see Figure C1) are found ($P_{1}$, $P_{2}$, $P_{3}$) and their equivalent viewpoint coordinate are found in the camera space ($C_{1}$, $C_{2}$, $C_{3}$). Any viewpoint coordinate, $C_{t}$ ($u_{t}$, $v_{t}$) can be expressed as the sum of two vectors:

(C1) $$\begin{array}{ccc} {\bar{C}}_{t}=a{\bar{C}}_{2}+b{\bar{C}}_{3},\;where\;\;{\bar{C}}_{2} & = & C_{2}-C_{1},\\ {\bar{C}}_{3} & = & C_{3}-C_{1},\\ {\bar{C}}_{t} & = & C_{t}-C_{1}. \end{array}$$

The values of a and b can be found as

(C2) $$b=\frac{{\bar{v}}_{t}-{ū}_{t}{\bar{v}}_{2}/{ū}_{2}}{{\bar{v}}_{3}{ū}_{3}{\bar{v}}_{2}/{ū}_{2}}$$

and

(C3) $$a=\frac{{\bar{v}}_{3}-b{ū}_{3}}{{ū}_{2}}.$$

This allows an equivalent point in robot space to be found as

(C4) $$\begin{array}{ccc} {\bar{P}}_{t} & = & P_{t}-P_{1}\\ & = & a{\bar{P}}_{2}+b{\bar{P}}_{3}. \end{array}$$

Such that the point $C_{t}$ transformed into robot space can be calculated by

(C5) $$P_{t}=P_{1}+a{\bar{P}}_{2}+b{\bar{P}}_{3}.$$
